# P-2172. Development of a Point-of-Care Diagnostic for Congenital Cytomegalovirus

**DOI:** 10.1093/ofid/ofae631.2326

**Published:** 2025-01-29

**Authors:** Chandler H Monk, Kevin J Zwezdaryk

**Affiliations:** Tulane University, New Orleans, Louisiana; Tulane University, New Orleans, Louisiana

## Abstract

**Background:**

Cytomegalovirus (CMV), is the most common congenital infection in the United States. Congenital CMV (cCMV) affects 3-6 per 1000 live born infants yearly. Up to 80% of cCMV cases are asymptomatic but can result in long term sequelae, including sensorineural hearing loss. Despite improved diagnostic strategies and therapeutics, universal screening programs are not standard care. Our laboratory is addressing this gap by designing a novel isothermal CRISPR-based point-of-care assay for cCMV. Preliminary studies using laboratory samples and conditions have demonstrated the feasibility of our approach.

**Methods:**

A CRISPR-Cas12a CMV specific detection system was designed against the CMV gene UL123 (immediate-early gene 1; IE1). Briefly, CMV was spiked into nuclease-free water samples and DNA was isolated using commercial kits. DNA was then amplified using standard PCR techniques. Amplified viral DNA was added to a reaction containing CRISPR-Cas12a, a fluorescent detection probe, and CMV UL123 guide RNA. The reaction was incubated at 37°C for 20 minutes. Fluorescent intensity of CMV spiked samples was compared to a no template control. The positive cutoff was calculated using the mean of the no template control + 3*SD.

**Results:**

Using this strategy, we observed a limit of detection at 10 infectious units/mL of CMV. There was no cross reactivity to other alpha- or betaherpesviruses. This was repeated using CMV-spiked human serum, saliva, and urine samples, which resulted in a similar limit of detection. To further improve our assay, we replaced the DNA isolation and PCR steps with an isothermal one-pot reaction, using a UL123 padlock probe based rolling circle amplification system. Employing this strategy, we can detect CMV in samples in 2 hours at 37°C, without the need for DNA isolation, prior amplification, or the use of PCR equipment. This approach was validated by designing targets to detect Epstein Barr Virus (EBV).

**Conclusion:**

We continue efforts to validate our isothermal point-of-care diagnostic to encourage cCMV universal screening implementation.

**Disclosures:**

All Authors: No reported disclosures

